# The influence of patient-centered teaching on medical students’ stigmatization of the mentally ill

**DOI:** 10.3205/zma001628

**Published:** 2023-06-15

**Authors:** Anna Hopp, Stefanie Dechering, Stefan Wilm, Markus Pressentin, Tobias Müller, Peter Richter, Ralf Schäfer, Matthias Franz, André Karger

**Affiliations:** 1 Heinrich-Heine-Universität Düsseldorf, Medizinische Fakultät, Düsseldorf, Germany; 2 Universitätsklinikum Düsseldorf, Centre for Health and Society, Institut für Allgemeinmedizin, Düsseldorf, Germany; 3 LVR-Klinik Düsseldorf, Klinik für Psychosomatische Medizin und Psychotherapie, Düsseldorf, Germany; 4 Universitätsklinikum Düsseldorf, Klinisches Institut für Psychosomatische Medizin und Psychotherapie, Düsseldorf, Germany

**Keywords:** medical education, bedside teaching, stigmatization, mentally ill, self-esteem

## Abstract

**Aim:**

Stigmatization by healthcare workers poses a challenge to providing care to the mentally ill. Bedside teaching during undergraduate medical education offers students an opportunity to directly interact with patients with a range of psychiatric disorders and thereby gather reflective experience. The present study investigates if this *supervised contact with mentally ill patients during a one-week clinical course on psychosomatic medicine leads to stigma reduction in medical students*. The factors influencing stigmatization were also investigated.

**Method:**

This was a prospective, non-randomized, controlled interventional study done in the 2019/20 winter semester involving fourth-year medical students who attended a week-long practical block on psychosomatic medicine (intervention group). This group was compared to students who had attended a week-long practical block with a somatic focus during the same time period (control group). Stigmatization was measured before and immediately upon completion of the week using the MICA-4 scale. Data on age, sex, experience with the mentally ill, interest in psychiatry/psychosomatics, and sense of self-worth were also gathered prior to starting the practical block. Analysis of the sample of 143 students with a complete basic data set was carried out using mixed ANOVA, multiple linear regression and moderator analysis.

**Results:**

In the context of clinical teaching with psychiatric patients, the stigmatization of the mentally ill among medical students decreased significantly more in the intervention group compared to the students in the control group who received instruction on somatic topics (*p*=.019, *η*^2^*_p_*=.04). In addition, being female, having previous experience with the mentally ill and general interest in the subjects of psychiatry or psychosomatics at T_0_ associated with lower stigma. In contrast, stigmatization was increased at the beginning of the study in males and those with low self-esteem. A moderating effect of the factors on stigma reduction was not seen.

**Conclusion:**

Undergraduate clinical instruction that enables direct contact and reflective experiences with the mentally ill leads to a reduction in the stigmatizing attitudes held by medical students toward the mentally ill. This underscores the need to have practical clinical instruction using patients.

## Zusammenfassung

**Zielsetzung:**

Stigmatisierung durch Mitarbeitende im Gesundheitswesen ist eine Herausforderung in der Versorgung psychisch kranker Menschen. Der Unterricht am Krankenbett in der medizinischen Ausbildung bietet die Möglichkeit, durch direkten Kontakt reflektierte Erfahrungen im Umgang mit Kranken diverser psychosomatischer Krankheitsbilder zu machen. In der vorliegenden Studie wurde untersucht, ob der *angeleitete Kontakt zu psychisch Kranken in einer einwöchigen klinischen Lehrveranstaltung der psychosomatischen Medizin bei Medizinstudierenden zu einer Reduktion der Stigmatisierung führt.* Zusätzlich wurden Einflussfaktoren der Stigmatisierung untersucht.

**Methodik:**

Dies erfolgte anhand einer prospektiven, nicht-randomisierten, kontrollierten Interventionsstudie im Wintersemester 2019/20 bei Medizinstudierenden im 4. Studienjahr, die an einer Praxisblockwoche der psychosomatischen Medizin teilnahmen (Interventionsgruppe). Verglichen wurde diese mit Studierenden, die im gleichen Zeitfenster eine Praxisblockwoche mit somatischem Schwerpunkt absolvierten (Kontrollgruppe). Hierbei wurde Stigmatisierung vor Beginn und unmittelbar nach Ende der Woche mittels der MICA4-Skala erhoben. Zusätzlich wurden vor Beginn Alter, Geschlecht, Erfahrung mit psychisch Kranken, Interesse am Fachgebiet Psychiatrie/Psychosomatik sowie Selbstwertgefühl erfragt. Die Auswertung der Stichprobe von 143 Studierenden mit vollständigem Basis-Datensatz erfolgte mittels Mixed-ANOVA, multipler linearer Regression und Moderatoranalyse.

**Ergebnisse:**

Im Rahmen des klinischen Unterrichts mit psychisch Kranken in der Interventionsgruppe nahm die Stigmatisierung psychisch Kranker durch Medizinstudierende im Vergleich zu Unterricht in somatischen Fächern der Kontrollgruppe signifikant stärker ab (*p*=.019, *η*^2^*_p_*=.04). Zudem gingen zu T_0_ weibliches Geschlecht, Vorerfahrung mit psychisch Kranken sowie allgemeines Interesse an den Fächern Psychiatrie oder Psychosomatik mit einer niedrigeren Stigmatisierung einher. Hingegen war Stigmatisierung bei männlichem Geschlecht und geringem Selbstwertgefühl zu Beginn der Untersuchung erhöht. Ein moderierender Effekt der Faktoren auf die Reduktion der Stigmatisierung zeigte sich nicht.

**Schlussfolgerung:**

Klinischer Unterricht in der medizinischen Ausbildung, der direkten Kontakt und reflektierte Erfahrungen mit psychisch Kranken ermöglicht, führt bei den Studierenden zur Reduktion einer stigmatisierenden Haltung gegenüber psychisch Kranken. Dies unterstreicht die Notwendigkeit eines praxisnahen klinischen Unterrichts unter Einbezug von Patient*innen.

## 1. Introduction

In Germany psychiatric disorders rank among the four most important causes for lost healthy years [1], [https://www.dgppn.de/schwerpunkte/zahlenundfakten.html, cited 2023 Jan 20]. Despite this, each year only 18.9% of those with mental health disorders in Germany receive general medical services or contact specialists [2]. In addition to structural barriers and public stigma, patients often described the experience of stigmatization by healthcare workers (iatrogenic stigma) as one of the causes for this underprovision of care [3], [4], [5]. Stigmatization also leads to self-stigma and an increase of suicidality in those affected [6], [7].

The term “stigmatization”, from the Greek word stigma (δτίγμα, “brand”), goes back to the labeling theory put forth by the sociologist Goffman and describes a process through which others assign people or groups to negatively connotated categories [8]. Factors with potential influence on the stigmatization of the mentally ill are identified in the literature. According to the literature, direct contact with the mentally ill specifically reduces stigmatization and is used in many anti-stigma campaigns [9], [10], [11], [12], [13]. Other stigma-reducing factors are female sex, a mentally ill relative or friend, and interest in psychiatry or psychosomatics [10], [14]. Contrary to this, stigmatization increases with male sex and little knowledge of mental illness [14], [15], [16]. Some authors postulate that a low sense of self-worth promotes the degradation of the mentally ill [17]. 

Stigma prevention is thus an important component in all healthcare education (also in post-graduate education and post-licensure training). The National Competency-based Catalogue of Learning Objectives in Undergraduate Medical Education (NKLM, version 2.0) states the necessity of recognizing and combatting stigma in the learning objectives VII.1a-20.2.4, VIII.2-05.2.2 and VIII.2-03.2.6 [https://nklm.de/zend/menu, cited 2021 Aug 6]. In the model medical degree program at the Heinrich Heine University Düsseldorf (HHU), students take many one-week-long practical blocks offered at different clinics in the Düsseldorf University Hospital (UKD) and at academic teaching hospitals and medical practices. Each practical block focuses on bedside teaching, patient consultations and case presentations (point-of-care learning and teaching) and brief clinical assessments (Mini-CEX) and case conferences [https://www.medizinstudium.hhu.de/en/duesseldorf-curriculum-of-medicine/practical-training, cited 2021 Aug 17]. During the fourth year of study, all of the students take a practical block on psychosomatic medicine (see attachment 1 ). In contrast to the practical blocks with instruction on somatic diseases, students attending the psychosomatic medicine block interact with simulated and real psychiatric patients in that they hold consultations with these patients and observe as others do the same. The simulated cases cover the clinical pictures of PTSD, depression and somatic symptom disorders taught in small groups [https://www.medizin.hhu.de/en/studying-and-teaching/institutions-and-programmes/skills-lab/courses/comed, cited 2022 Dec 28]. Contact with real patients entails a range of clinical pictures within psychosomatic medicine and takes place on the different wards of the Clinic for Psychosomatic Medicine and Psychotherapy. The students are given the opportunity to directly interact with the mentally ill (“learning at the point of care”), in addition to observing doctor/patient interactions (e.g., group therapy sessions, bedside teaching). Afterward, students work through these simulated and real cases in the form of a detailed report, theoretical input (including parallel lectures) and feedback (from the group and simulated patients) in order to explore mental illness and psychiatric disease in a case-based manner and acquire the appropriate clinical skills. In addition to this, the cases are presented to the group and followed by discussion of the experiences and the knowledge gained (case conference).

This study investigated if supervised contact with mentally ill patients during a one-week clinical course on psychosomatic medicine resulted in a reduction of stigmatization in medical students. The factors listed as having an influence on stigma were also investigated.

The individual hypothesis were:

- H_1_: Over the time period there is a significantly stronger reduction of stigmatization in the intervention group (IG) compared to the control group (CG).
- H_2_: The predictive variables of sex, interest in psychiatry/psychosomatics, presence of mental illness in the family or circle of friends, and sense of self-worth will predict the extent of stigma in the IG and CG at the beginning of the week-long practical block.
- H_3_: The factors identified above moderate a reduction in stigmatization in the IG.

## 2. Methods

### 2.1. Study design

A prospective, controlled, non-randomized, pseudonymized interventional study was carried out at the HHU Medical Faculty to test these hypotheses. A positive ethics vote was given by the Medical Faculty’s Ethics Commission (study no.: 2019-466).

As part of the study, the change in medical students’ stigmatization of the mentally ill was compared between the IG and CG. To do this, the level stigmatization was measured in both groups using the MICA-4 scale (see section 2.3) and compared for potential changes during the week-long period and between the IG and CG. A mixed ANOVA (between-within) was applied as the statistical design.

The potential predictors of age, sex, presence of mental illness in the family or circle of friends and acquaintances (*PsychFam*) and general interest in the field of psychiatry/psychosomatics (*GenInt*) were measured using a questionnaire; the sense of self-worth was measured using the Rosenberg scale (both at T_0_ only; see section 2.3). The number of patient interactions in the week per medical student were recorded at T_1_.

Multiple linear regression and moderator analysis were carried out to analyze the degree of the influence exerted on the stigmatization.

### 2.2. Sample recruitment

Only fourth-year medical students enrolled at the HHU and capable of giving consent were included in the study. The data was collected during the 2019/20 winter semester. Students who attended the practical block on psychosomatic medicine were assigned to the IG. Students who attended a practical block with a focus on somatics (practical blocks on cardiology, urology, gastroenterology, ophthalmology) were assigned to the CG. In contrast to the instruction given to the CG, the IG experienced supervised contact with mentally ill patients and explored psychiatric disorders in more depth based on patient cases (see Introduction). While the practical blocks included in the CG had instruction on somatic disease pictures, there were no guided interactions with *mentally* ill patients.

Recruitment took place within the scope of the practical blocks at the Düsseldorf LVR Clinic, in the Clinic for Psychosomatic Medicine and Psychotherapy and at the Düsseldorf University Hospital in the Clinic for Cardiology, Pneumology and Angiology, the Clinic for Gastroenterology, Hepatology and Infectious Diseases, the Clinic for Urology, and the Clinic for Ophthalmology. The data was collected on paper in the seminar rooms at each clinic after the students had received an informational email on the friday before and after detailed information was given in-person at the clinics and the students had submitted written consents to voluntarily participate in the study. Non-participation had no negative repercussions for the students; likewise, the consent to participate could be revoked at any time.

The exclusion criteria were prior attendance of a practical block on psychosomatic medicine or psychiatry and retrospectively an attendance rate of the current practical block lower than 70%. In addition, students in the IG and CG were subsequently excluded if they had not been present to observe a patient consultation and had not conducted one themselves.

A sample size of *N*=200 students for the mixed ANOVA was calculated using G*Power with an effect size of ƒ=.1, a significance level α=.05, and a desired power level of at least 1-*β*=.80 [18].

**Figure 1:**
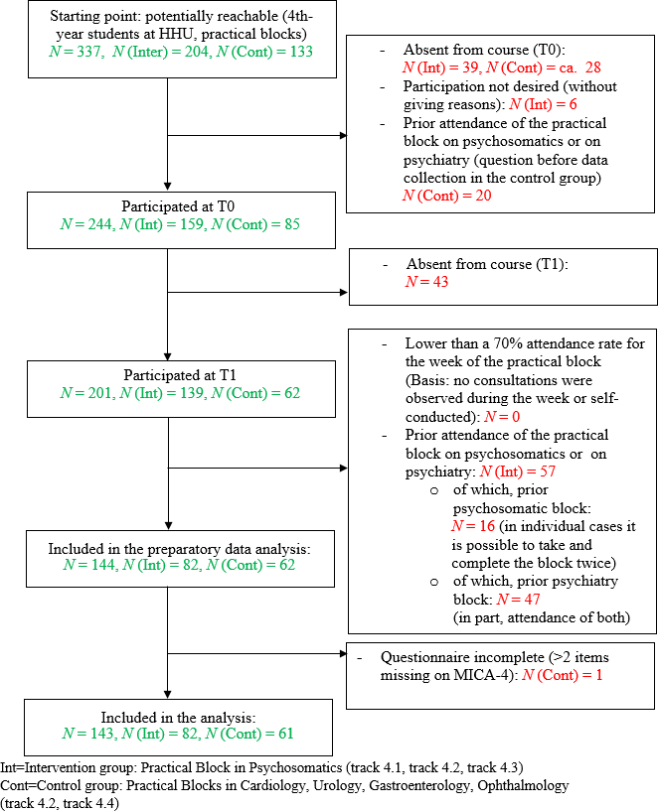
Consort flow chart: Subject recruitment

Figure 1 (Fig. 1) reports the sample generation and final composition of the groups.

### 2.3. Measuring instruments

In addition to two validated survey instruments, a self-generated questionnaire was used (see attachment 2 ).

The German version of the Mental Illness: Clinician’s Attitudes Scale 4 (MICA-4) was used to collect the data on stigmatizing attitudes [19], [20]. This entailed a 6-point Likert scale with 16 items on which the students rated their responses as 1=“completely agree” to 6=“completely disagree”. It must be noted that 10 items (1, 2, 4, 5, 6, 7, 8, 13, 14, 15) were inverted in the analysis. A total number of points was calculated as a sum of all of the points, whereby a high overall score means a more negative stigmatizing attitude (minimum points possible=16, maximum points possible=96). A study by Gabbidon et al. showed Cronbach’s α=72 for the English version [19]. 

The students' global self-worth was assessed using the Rosenberg scale [21]. This involves a 4-point Likert scale with 10 items. The scale ranges from 1=“strongly disagree” to 4=“strongly agree”. Items 2, 5, 6, 8 and 9 are inverted for the analyses. Here, too, the number of points are added up for a sum total, whereby a high value reflects a high self-esteem (minimum points possible=0, maximum points possible=30). For the revised German version of the scale, calculations showed Cronbach's α=85 and an item discrimination index of *r*=.55 on average [22]. The scale was used only once at T_0_ in terms of a trait component [23], [24].

The self-generated questionnaire (see attachment 2 ) covers information about the current practical block, age, sex and statements about mental illness among family/friends, and level of general interest in psychiatry/psychosomatics. It also contains questions to confirm the inclusion and exclusion criteria given above.

### 2.4. Statistical analysis

The analysis of differences between the groups regarding the factors of sex, PsychFam and GenInt was carried out using the chi-squared test; the analysis of differences regarding the factors of age, sense of self-worth and the variable stigma was carried out with the unpaired *t*-test. 

The test of hypothesis H_1_ was done for the dependent variable stigma using a mixed ANOVA with the between-subjects factor group assignment (IG and CG) and the within-subjects factor time (T_0_ and T_1_; time period T_0_ to T_1_: 5 days) and the overall MICA-4 score as dependent variable.

Hypothesis H_2_ was tested using multiple linear regression. After considering the necessary assumptions, a stepwise regression was carried out. Backward elimination was selected to minimize the risk of a type II error [25]. The overall MICA-4 score at T_0_ and T_1_ were considered dependent variables. After completing the stepwise regression, the variables which significantly contributed to explaining the variance were included in a hierarchical model. It must be noted that the factor of age did not show a linear relationship to the variable stigma and thus was not included in the regression analysis.

The moderator analysis was then carried out to test hypothesis H_3_. Here, group assignment was selected as independent variable and the difference between overall MICA-4 score at T_0_ and T_1_ as dependent variable. Sex, GenInt, PsychFam and self-esteem were put into the model as possible moderator variables.

The statistical analyses were performed using the statistics software SPSS, version 26, and Makro PROCESS, version 3.5, [http://www.processmacro.org/index.html, 2021 Jul 19].

### 2.5. Preparatory data analysis

#### 2.5.1. Missing values and deletion

In the data set of 144 students, there were three students who each had a missing value in the MICA-4 scale at T_0_. Single imputation was applied to complete the data set. The data set for one student was excluded from all of the analyses because seven items in the MICA-4 scale were missing at T_1_ [26] (see figure 1 (Fig. 1)). A total of seven students were excluded on a case basis from the regression analyses because they had not given any information about the variables PsychFam and/or GenInt.

#### 2.5.2. Outliers

When looking for outliers, two students stood out. One student in the CG showed an unusually high overall score (56 points) on the MICA-4 scale at T_0_ with 1.5-fold of the interquartile range (*IQR*) above the upper quartile (Q_3_). In the IG there was an especially low overall score (5 points) at T_0_ on the Rosenberg scale (Q_1_-1.5*IQR). The decision not to exclude was made in both cases [27].

A total of 143 students with a complete basic data set were included in the final analysis.

## 3. Results

### 3.1. Test subjects

The final sample with the complete basic data set was comprised of 51 male (36%) and 92 female subjects (64%). In regard to age distribution the mean was 24 years (see table 1 (Tab. 1)). Both correspond to the values for the entire student cohort at this semester level.

### 3.2. Descriptive results

In regard to stigmatization (MICA-4), there was an average overall score of 41.31 for the sample at the beginning of the practical blocks and of 40.01 at T_1_ (see table 2 (Tab. 2)). The students had an average of 23.38 points on the Rosenberg scale (self-esteem) (see table 2 (Tab. 2)). In both groups, the majority of subjects stated they did not have any mentally ill family members or acquaintances. By contrast, the responses to the question about general interest (T_0_) showed that the majority of students claimed to have a general interest in the subjects of psychiatry/psychosomatics (see table 2 (Tab. 2)).

### 3.3. Interferential statistical results

#### 3.3.1. Testing for differences in distribution

At the start of the week, the groups were not significantly different from each other in terms of stigmatization (MICA-4), self-esteem (Rosenberg scale) and the factors of sex, age, PsychFam and GenInt. Thus the starting conditions were the same for both groups (see table 3 (Tab. 3)).

#### 3.3.2. Test of hypothesis 1

**Figure 2:**
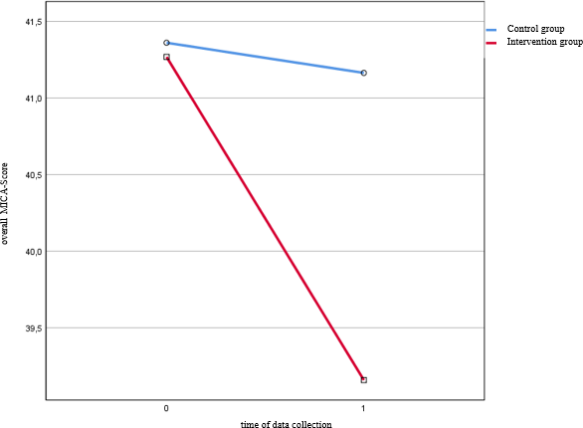
Results of the mixed ANOVA – reduction in stigmatization

When testing the veracity of H_1_ (mixed ANOVA), a statistically significant interaction between measuring time point and the two groups was seen (*p*=.019, *η*^2^*_p_*=.04) (see figure 2 (Fig. 2) and table 4 (Tab. 4)). This shows a medium effect size according to Gignac and Szodorai (r=2) [28]. Likewise, the main effect of the factor measuring time point was significant in the IG (*p*<.001, *η*^2^*_p_*=.152) (see table 4 (Tab. 4)). It is accordingly assumed that there is a significantly greater reduction of the overall MICA-4 score (stigmatizing attitude) in the medical students in the IG compared to the CG.

The result of the t-test is confirmed at T0 by the main effect of group assignment (section 3.3.1; see table 4 (Tab. 4)): At the beginning of the week, the groups did not differ significantly in terms of overall MICA-4 score. At T_1_ a tending difference in the overall MICA-4 score between the groups was detected (*p*=.068, *η*^2^*_p_*=.023). This means there was only a tendential difference between the IG and CG regarding the stigmatization of the mentally ill at the end of the week-long practical block (despite the significantly greater reduction in stigma in the IG compared to the CG during the week).

#### 3.3.3. Test of hypothesis 2

The following variables were significantly included in the regression model at T_0_: GenInt, PsychFam, sense of self-worth, and sex. The variable of group assignment was excluded based on its insignificant contribution to the explanation of variance (beta=-.005, *p*=.950). Factors which were included at T_1_ were PsychFam, GenInt, sense of self-worth, and group assignment. Excluded at T_1_ was the variable sex (beta=.132, *p*=.108).

In the hierarchical regression analysis (see table 5 (Tab. 5)) it was shown that the predictors of sex, GenInt, PsychFam and sense of self-worth significantly predict the extent of stigmatization regardless of the practical block (*p*<.001). The greatest influence here was exerted by general interest. Both students with a general interest in psychiatry/psychosomatics and those with a mentally ill relative or friend showed significantly lower overall MICA-4 scores and thus a more accepting attitude toward people with mental illness. The same was seen in the female students in contrast to their male counterparts. Likewise, students with low self-esteem showed significantly more frequent belittling of the mentally ill.

#### 3.3.4. Test of hypothesis 3

A significant moderating effect of the identified factors on the reduction of stigma during the week of clinical instruction could not be detected.

## 4. Discussion

### 4.1. Place in the literature

This study was able to demonstrate that students take on a more positive attitude toward the mentally ill during clinical instruction focused on psychosomatics compared to students in other (somatic) courses. This corroborates the results of many anti-stigma studies around the world [10], [11], [14]. In Germany this had previously only been analyzed for specific disorders such as schizophrenia and depression [29], [30]. Because the present study investigates the general stigmatization of the mentally ill without restricting the disease entity, it broadens German stigma research in regard to destigmatization in German medical education.

Furthermore, some empirical papers have shown that experiences in the personal sphere lead to greater acceptance of people with mental illness [9], [10], [11], [15]. This was the case in the present study, too: Students who have a relative or acquaintance with mental illness display a more positive attitude toward the mentally ill. In the sample here, the same reduced stigmatization of the mentally ill was seen in the students who had general interest in the field of psychiatry or psychosomatics. A study by Janoušková et al. had similar findings [14]. There is, at present, no visibly uniform pattern in the empirical research regarding the influence of the sociodemographic factor sex. In most cases it is reported that women have a less stigmatizing attitude toward the mentally ill than men [10], [11], [14], [15], [16]. The present study also confirms this. 

Moreover, the results suggest that people with a more negative self-regard have a greater tendency to look down on the mentally ill [17]. In terms of the* downward comparison theory* [31], [32], this association could possibly be explained in that one's self-esteem can be enhanced by diminishing or degrading others. However, no further empirical proof for this is currently available, making further studies necessary.

Based on the present study, it was possible to explore for the first time how the identified factors affect the extent of the prevailing stigmatizing attitudes, but not changes in them. Further studies are needed due to the gap in the literature.

### 4.2. Limitations

First, it must be noted that the previously calculated number of cases, N=200 subjects, was not met. Nevertheless, based on the final sample size of 143 subjects, significant results with a medium effect size were achieved in the analysis of the primary outcome (interaction effect in mixed ANOVA). Furthermore, other studies have yielded comparable results with a similar sample size [11].

The representative nature of the results with no randomization is potentially limited; however, randomization was not feasible in the context of regular curricular courses.

Also, it must be noted that there is no German study validating the MICA-4 scale. Since this scale appeared especially suitable for asking questions at the time of data collection and the English validation study presented satisfactory results, we made the decision to use it. According to the authors, the MICA-4 scale was translated in an appropriate manner (two translators, including subsequent reverse translation). In addition to this, we were also able to demonstrate a Cronbach's α of .66 in our reliability analysis at T_0_, which is similar to the reliability given in the validation study of the English version (α=.72) [19].

In regard to the assessment of the assumptions required for the analytical tests used, it is worth mentioning that the MICA-4 scale and the Rosenberg scale both entail ordinally scaled Likert scales, which according to some authors should be analyzed non-parametrically [33]. This view, however, is a controversial topic of discussion. Based on the current literature, the use of parametric designs for the analysis of overall scores is still of meaningful value [34], [35], [36], [37], so that we decided to use the* t*-test and the mixed ANOVA.

Furthermore, within the scope of the present study it is only possible to make a general statement regarding the effect of the complex intervention during the psychosomatic practical block on the stigmatization of the mentally ill by medical students. Although a significant reduction in stigmatization of the mentally ill was visible as a result of participation in the practical block, it is not clear what this specifically correlates with based on the collected data (see table 6 (Tab. 6)). Along with the supervised contact with patients (self-conducted consultations and observation of other consultations), the students worked through patient cases in detail and looked in depth at psychiatric disorders (see above). Each one of these separate components or combination thereof can ultimately have led to a change in attitude toward the mentally ill. A strict separation of the course sessions from each other was not done in our study. It is obvious that direct, concrete interaction enables empathy and compassion with a patient's history and suffering, making it possible to differentiate between stereotypes. However, this assumption cannot be concretely proven based on the present study. A systematic design with the assignment of separate elements within the IG (with and without patient contact, with and without theoretical context for the practical experiences) could yield more detailed information in future studies.

In addition to this, the stigmatization only tended to differ between the groups at the end of the practical block despite the different degrees of stigma reduction during the week; a significant difference was not visible yet at this point (see table 4 (Tab. 4)). During the fourth year of study, medical students at HHU also come into supervised contact with psychiatric patients in the practical block on psychiatry. It must be assumed that, as a result of this additional direct contact, stigmatization can be reduced even more. For this reason, it seems that a repetition of the study after completion of both practical blocks would be worthwhile.

## 5. Conclusions

As part of the clinical instruction in psychosomatic medicine in the Düsseldorf model degree program in medicine, future physicians come into supervised contact with patients and are given the opportunity to grapple in detail with psychiatric cases so that they can develop a more accepting attitude toward the mentally ill.

This emphasizes the need during medical education to have a sufficient measure of structured, practical contact with psychiatric diseases and disorders and thus continue to confront the problem of stigma, as called for in the NKLM.

## Competing interests

The authors declare that they have no competing interests. 

## Supplementary Material

Schedule for the Practical Block in Psychosomatic Medicine and Psychotherapy – Winter semester 2019/20

Questionnaire for the study: “Changes in medical students’ stigmatization of psychiatric patients as a result of direct patient contact (StigMed)”

## Figures and Tables

**Table 1:**
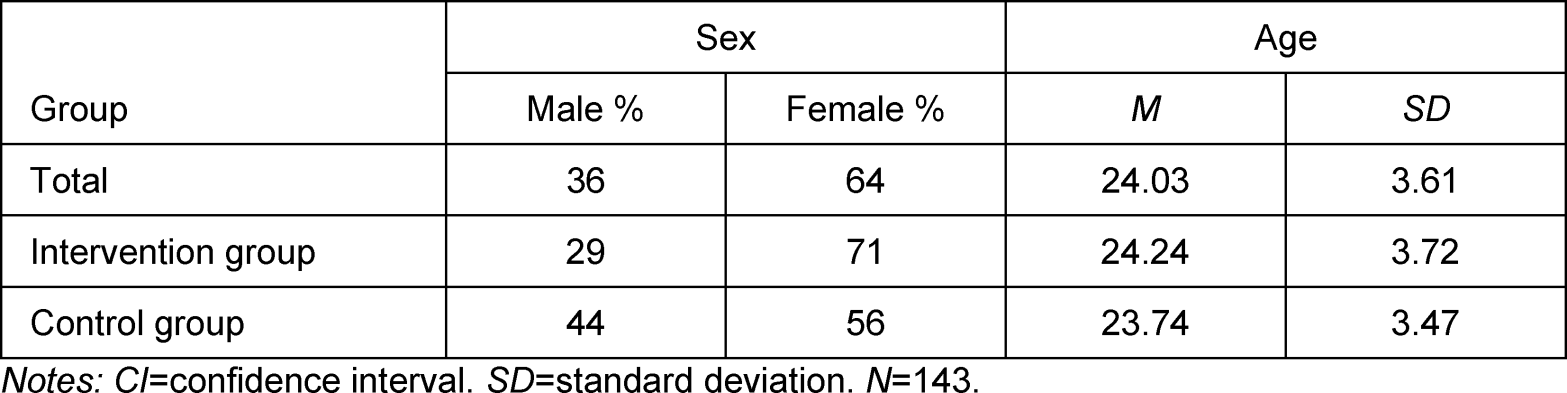
Sample composition regarding sex and age

**Table 2:**
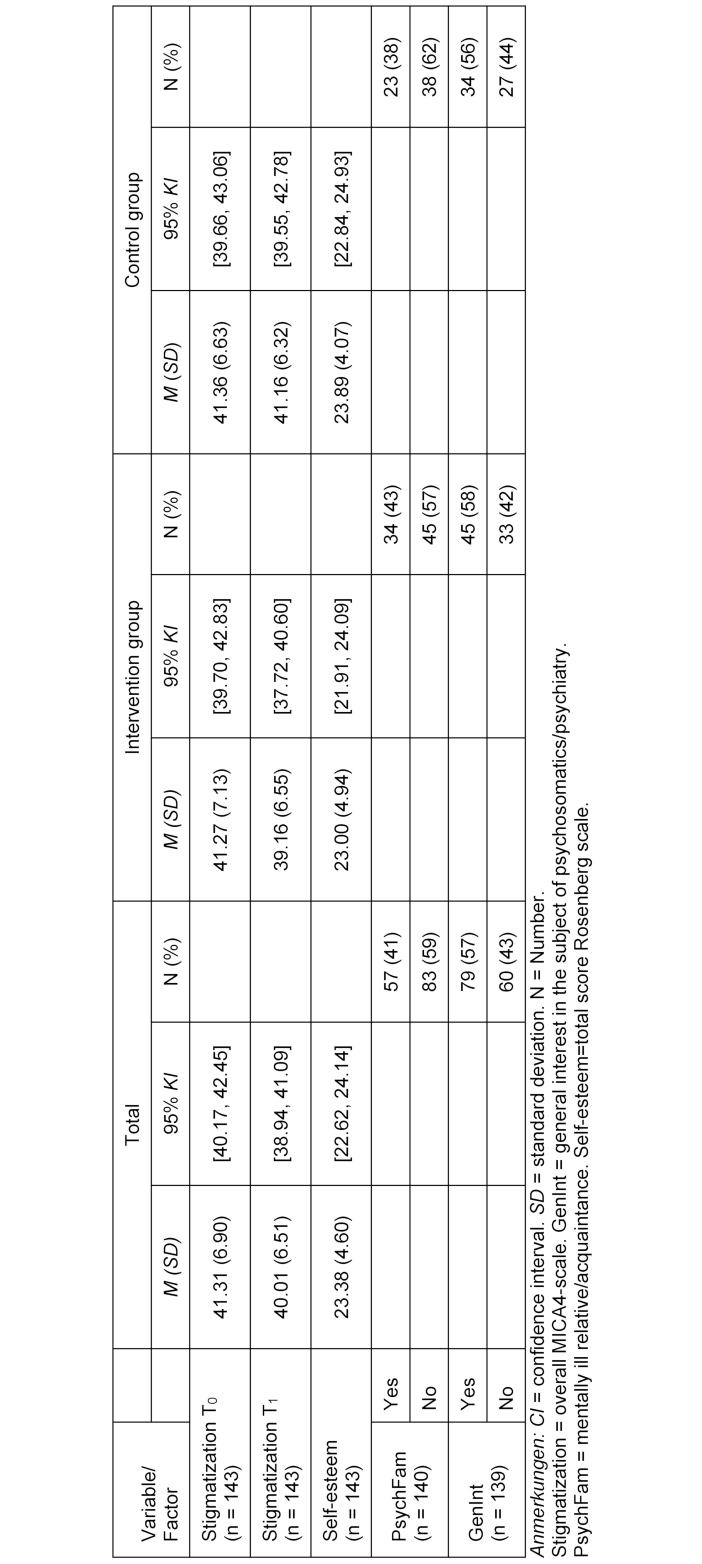
Descriptive results of the dependent variable stigmatization and the predictors of stigmatization

**Table 3:**
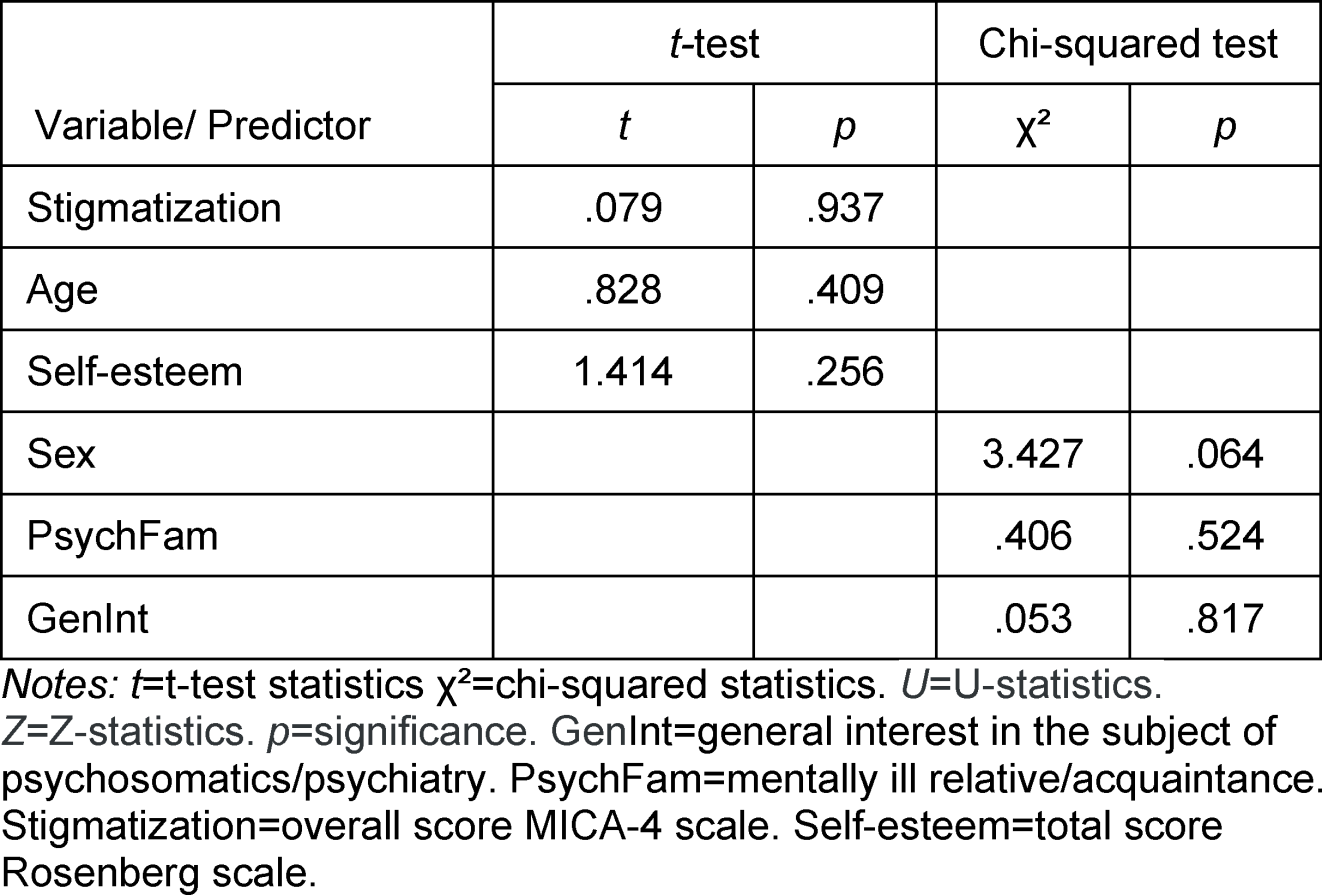
Results of the analysis for differences in response behavior between the intervention group and control group at T_0_

**Table 4:**
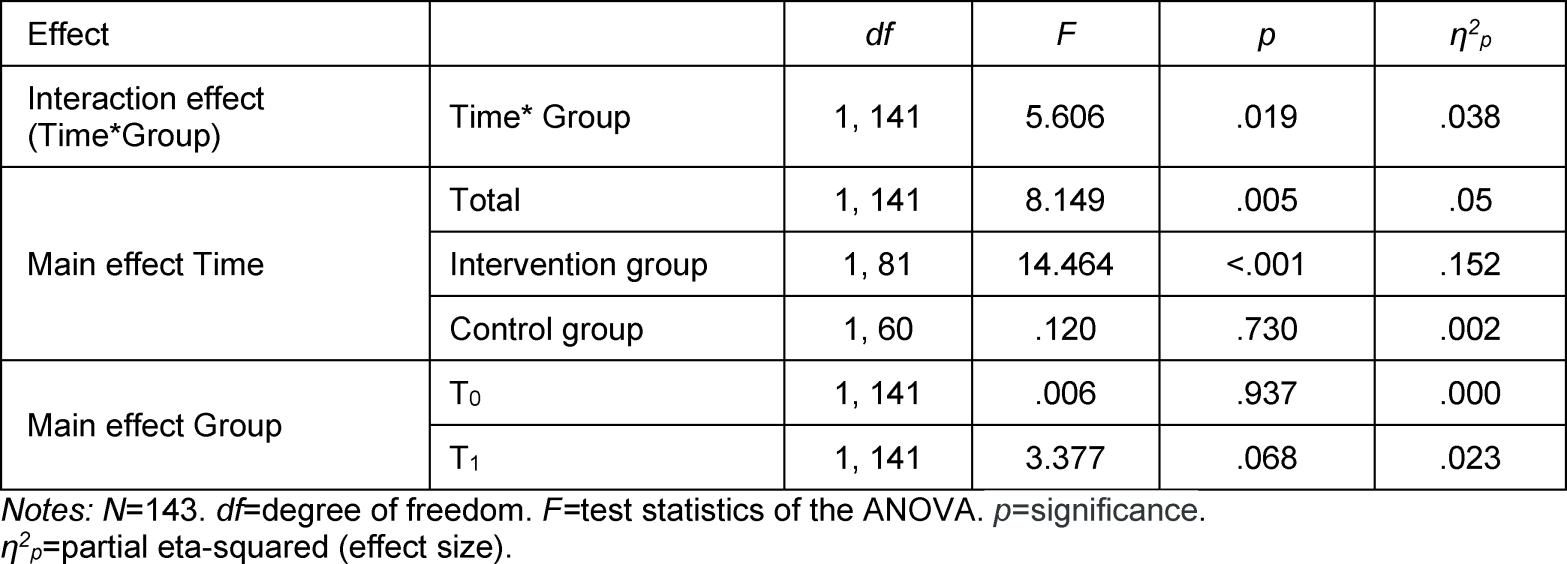
Results of the mixed ANOVA (H_1_ test)

**Table 5:**
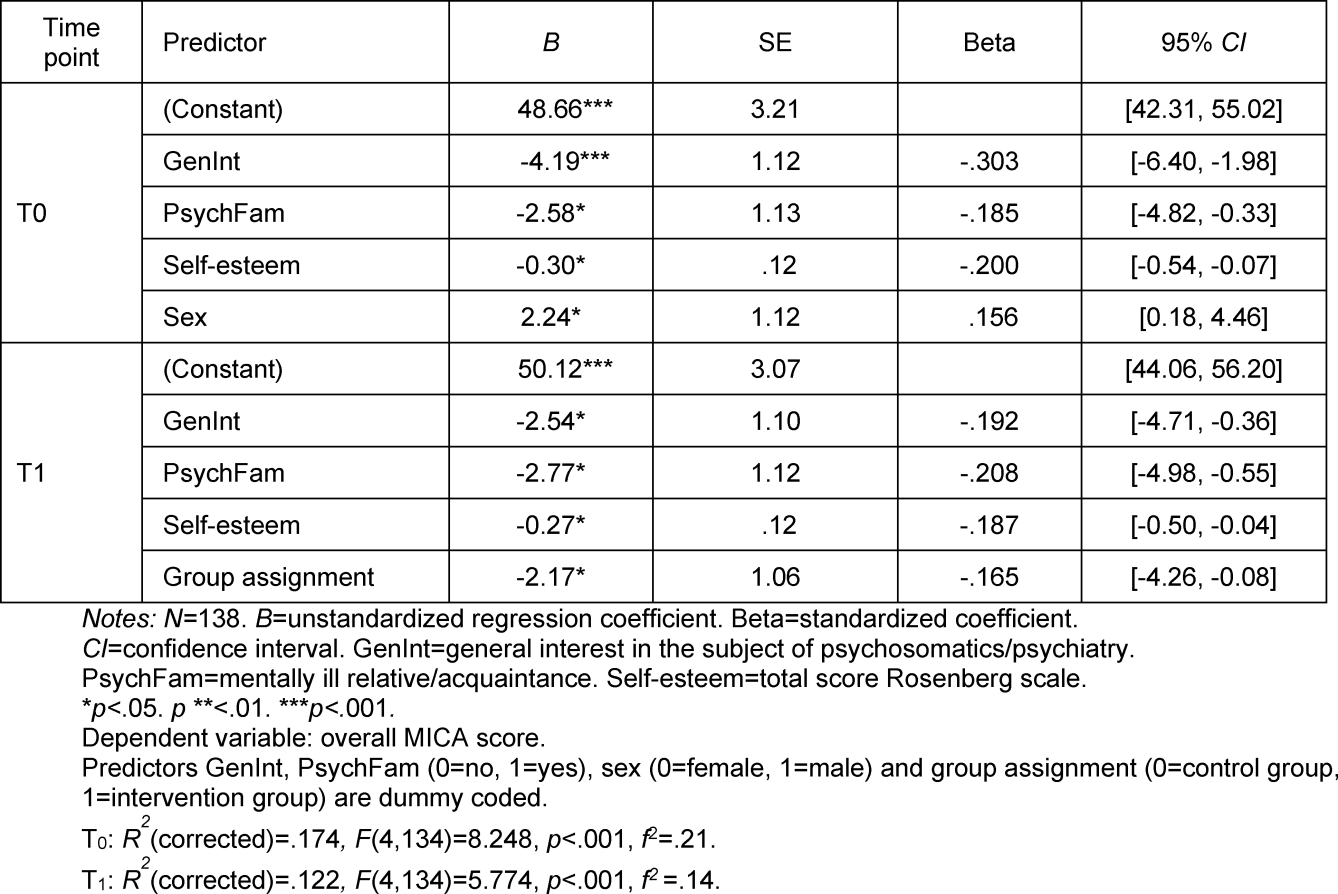
Results of the multiple linear regression regarding the influence of factors on the stigmatization of the mentally ill

**Table 6:**
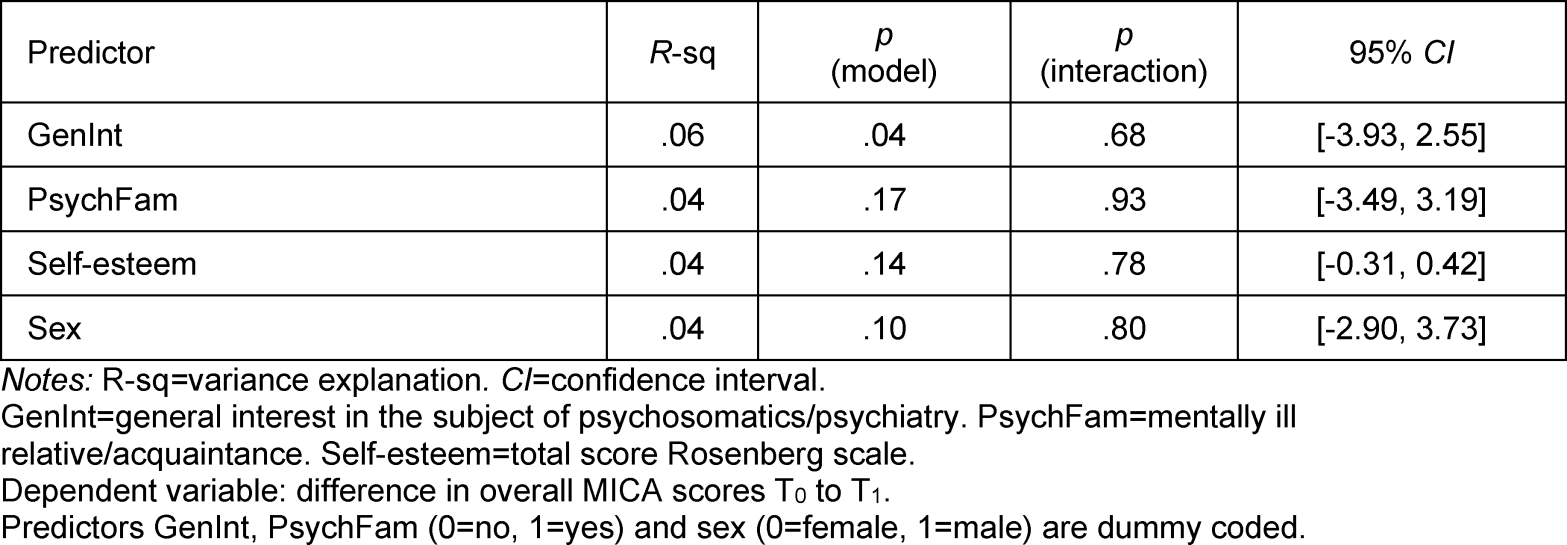
Moderator analysis regarding the influential factors on stigma reduction
