# Supplementary material for: The influence of patient-centered teaching on medical students’ stigmatization of the mentally ill
Source: GMS J Med Educ. 2023 Jun 15;40(4):Doc46. doi: 10.3205/zma001628 (PMC10407581; doi:10.3205/zma001628)
Supplement: Schedule for the Practical Block in Psychosomatic Medicine and Psychotherapy – Winter semester 2019/20 [file JME-40-46-s-001.pdf]

Attachment 1: Schedule for the Practical Block in Psychosomatic Medicine and Psychotherapy – Winter semester 2019/20

Monday	Tuesday	Wednesday	Thursday	Friday
8:10 - 9:45 a.m.<br><br>Introduction to the week<br><br>T_0	8:00 – 10:30 a.m.<br><br>Reading cases and conducting patient consultations (*Learning at the point of care*)	Elective subjects	8:15 a.m. -1:30 p.m.<br><br>3 *bedside teaching* sessions with simulated patients (CoMeD)<br><br>including Mini-CEX assessments	8:15 – 9:50 a.m.<br><br>Brief presentation of patient cases (*learning at the point of care*)
10:00 – 11:00 a.m.<br><br>*Bedside teaching* based on a patient consultation<br><br>Preliminary discussion on *learning at the point of care*				10:00 – 11:00 a.m.<br><br>Participation in group therapy session (*bedside teaching*)
11:00 – 11:30 a.m.<br><br>Getting patients on the ward ready for the *learning at the point of care*	11:00 a.m. – 12:30 p.m.<br><br>Case conference			11:30 a.m. – 1:00 p.m.<br><br>Case conference, including a review of the week<br><br>T_1
2:00 – 4:00 p.m.<br><br>Lectures	2:00 – 4:00 p.m.<br><br>Lectures		2:00 – 4:00 p.m.<br><br>Lectures	2:00 – 4:00 p.m.<br><br>Lectures

Attachment 1 to Hopp A, Dechering S, Wilm S, Pressentin M, Müller T, Richter P, Schäfer R, Franz M, Karger A. *The influence of patient-centered teaching on medical students' stigmatization of the mentally ill.* GMS J Med Educ. 2023;40(4):Doc46. DOI: 10.3205/zma001628

Notes:

MiniCEX = Mini Clinical Examination, *CoMeD* = Communication in Medical Education Düsseldorf

Time point of data collection on "The influence of patient-centered teaching on medical students' stigmatization of the mentally ill, StigMed":
$\mathbf{T_0}$= Time point of first questionnaire, $\mathbf{T_1}$= Time point of second questionnaire

The lectures are held according to a separate lecture schedule. More (current) information can be found here:
https://www.medizinstudium.hhu.de/duesseldorfer-curriculum-medizin/themen-und-studienbloecke/kopf-und-nervensystem, cited 2023 Jan 23
