# Supplementary material for: The influence of patient-centered teaching on medical students’ stigmatization of the mentally ill
Source: GMS J Med Educ. 2023 Jun 15;40(4):Doc46. doi: 10.3205/zma001628 (PMC10407581; doi:10.3205/zma001628)
Supplement: Questionnaire for the study: “Changes in medical students’ stigmatization of psychiatric patients as a result of direct patient contact (StigMed)” [file JME-40-46-s-002.pdf]

Attachment 2: Questionnaire for the study:
"Changes in medical students' stigmatization of psychiatric patients as a result of direct patient contact (StigMed)"

To start, please create your own participant code (*pseudonymization*):

Second letter of your mother's first name	First letter of your father's birth month	The number of your father's birth month	Last letter of your birthplace

Age: ____________

Gender: ☐ female ☐ male. ☐ ____________

Current practical block: ☐ Psychosomatics ☐ Urology ☐ Cardiology ☐ Gastroenterology

Practical Block on Psychosomatics already completed? Yes ☐ No ☐

Practical Block on Psychiatry already completed? Yes ☐ No ☐

Mentally ill person in your family/circle of acquaintances: Yes ☐ No ☐

General interest in the subject of psychiatry or psychosomatics: Yes ☐ No ☐

To be filled out at the end of the week-long practical block (T1):

Number of patient consultations for which I was present during this practical block week (including consultations with simulated patients):

☐ 0 ☐ 1 ☐ 2 ☐ 3 ☐ 4 ☐ >4

Number of patient consultation which I conducted myself during this practical block week (including consultations with simulated patients):

☐ 0 ☐ 1 ☐ 2 ☐ 3 ☐ 4 ☐ >4

Attachment 2 to Hopp A, Dechering S, Wilm S, Pressentin M, Müller T, Richter P, Schäfer R, Franz M, Karger A. *The influence of patient-centered teaching on medical students' stigmatization of the mentally ill.* GMS J Med Educ. 2023;40(4):Doc46. DOI: 10.3205/zma001628
